# Comparison of physicochemical properties, amino acids, mineral elements, total phenolic compounds, and antioxidant capacity of Cuban fruit and rice wines

**DOI:** 10.1002/fsn3.2328

**Published:** 2021-06-01

**Authors:** Lázaro Núñez, María P. Serratosa, Ana Godoy, Laura Fariña, Eduardo Dellacassa, Lourdes Moyano

**Affiliations:** ^1^ Department of Agricultural Chemistry, Soil Science and Microbiology Faculty of Sciences Universidad de Córdoba Córdoba España; ^2^ Food Science and Technology Department Faculty of Chemistry Montevideo Uruguay

**Keywords:** amino acids, antioxidant capacity, Cuba, grape wine, mineral elements, rice wine, tropical fruit wine

## Abstract

Physicochemical characterization, amino acids contents, minerals composition, total phenolic compounds, and antioxidant capacity of Cuban wines from different raw materials were studied. The wines studied were grape wines, tropical fruit wines, and rice wines. Twenty‐one amino acids were identified and quantified, being Asp and Glu detected in all wines. The highest concentration of total amino acid content was found in wines elaborated from Cimarrona grape subjected to maceration with grape skins, while the raisined mixture grape wine presented the lowest values, probably caused by the amino acid degradation during the dehydration process by sun exposure. Minerals quantified were range amount limits of acceptable according to the OIV recommendation. Total phenolic compounds and antioxidant capacity showed the greatest values in wine from roasting rice. No statistical separation could be clearly observed by multivariate principal component analysis; however, 3 wine groups could be defined taking account the scores on the PC1.

## Graphical abstract

Physicochemical properties, amino acids, mineral elements, total phenolic compounds, and antioxidant capacity of 10 Cuban wines elaborated from different raw materials.

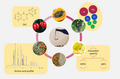

## 1 INTRODUCTION

Winemaking is one of the most ancient of man's technologies and is now one of the most commercially prosperous biotechnological processes (Moreno‐Arribas & Polo, 2005). In this sense, in the last two decades, due to the generation of surplus production and over‐ripen fruits, several non‐grape fruits are used in many parts of the world for the production of wine, such as raspberry, cherry, strawberries, pawpaw, banana, watermelon, mango, guava, kiwi, carambola, apple, plum, peach, and wild apricot (Duarte et al., 2010; Ogodo et al., 2015, 2018; Sevda et al., 2011; Vidya & Neela, 2004). Moreover, rice wine is a Chinese traditional fermented alcoholic beverage for more than 5,000 years, used in traditional medicine due to their beneficial effects, and today, it is one of the most popular alcoholic beverages. In the brewing process of rice wine, rice starch is converted to glucose by koji, a grain (e.g., rice, wheat) cultivated with mold (e.g., *Aspergillus oryzae*), and glucose is converted to ethanol by yeast (Koda et al., 2012; Wu, Xu, Long, Wang, et al., 2015; Wu, Xu, Long, Zhang, et al., 2015).

These wines are an important source of bioactive compounds highlighting phenolic compounds, amino acids, minerals, vitamins, and carotenoid pigments. Most bioactive compounds present in the fruit and rice are bonded to insoluble plant compounds, and during the winemaking process, many of them are liberate and can be transferred into the wine, reducing the risks of certain diseases (Amidžić et al., 2016; Pantelic et al., 2014; Shahidi, 2009; Shen et al., 2010, 2011; De Souza et al., 2018; Zhong et al., 2020). Among the different bioactive compounds studied in these type wines, phenolic compounds present an important antioxidant capacity by different ways: reducing agents, hydrogen donators, free radical scavengers, singlet oxygen quenchers, and so forth (Mundaragi & Thangadurai, 2018; De Souza et al., 2018). Also, daily consumption of wine in moderate quantities contributes signiﬁcantly to the requirements of human organism for essential mineral elements. But, special attention must be given to trace elements for their potential toxicity (OIV, 2020; Plotka‐Wasylkaa et al., 2017; Zuñiga et al., 2014). Moreover, amino acids are important both as essential components of proteins and for their roles in energetic metabolism, neurotransmission, and lipid transport (Shen et al., 2010). Also, are precursors for aroma compounds and directly contribute to wine's aroma, taste, and appearance. For wines elaborated with different raw materials, the amino acid proﬁles vary according to origin, wine‐making technique, and variety. In this sense, the studies about amino acid composition may be considered as a useful tool to ensure the authenticity of fruit and rice wines (Shen et al., 2010; Valero et al., 2003).

For a long time, Cuba has handcrafted fermented beverages from a wide variety of raw materials such as tropical fruits, pumpkin, grape, corn, and rice. In recent years, and mainly due to the creation of the National Coordinator of Wine Clubs of Cuba (NCWCC), Cuba has become one of the countries that produce wines from a greater range of raw materials reaching a great international interest. Although the number of publications about fruit wines and rice wines has increased in recent years (Chay et al., 2020; Kosseva et al., 2017; Płotka‐Wasylkaa et al., 2018; Qian et al., 2019; Seixas et al., 2020; De Souza et al., 2018; Sun et al., 2020; Velic et al., 2018; Wu, Xu, Long, Wang, et al., 2015; Wu, Xu, Long, Zhang, et al., 2015; Zhong et al., 2020), no investigation has been conducted to appraise health‐related major components of these Cuban wines. This study, therefore, evaluates the bioactive‐nutritional compounds (mineral elements, amino acids, and total polyphenols content), antioxidant activities (DPPH and ABTS), and the correlation between the selected factors and the samples of Cuban wines with the objective to highlight these unknown wines for production of healthier wine options.

## 2 MATERIALS AND METHODS

### 2.1 Wine samples

Ten types of wines elaborated with different winemaking processes and diverse raw materials, Aramon grapes (*Vitis vinífera* L. cv. Aramon), Cimarrona grapes (*Vitis tiliifolia*), frutilla (*Pereskia aculeata*, *Plantae*, *Cactaceae*), papaya (*Carica papaya* L., *Plantae*, *Caricaceae*, cv. Maradol), carambola (*Averrhoa carambolo* L, *Plantae*, *Oxalidaceae*), *and* rice (*Oryza sativa* L., *Plantae*, *Poaceae*) were selected for this study from Camagüey province, Cuba. The five grape wines (G) were G1 (Aramon grape subjected to maceration with grape skins), G2 (Cimarrona grape subjected to maceration with grape skins), G3 (Aramon grape with *Botrytis cinerea* “noble rot” and wine aging with *Rhizophora mangle* L. chips), G4 (mixture of Aramon grape and Cimarrona grape with *Botrytis cinerea* “noble rot” and wine aging with *Rhizophora mangle* L. chips), and G5 (mixture of raisined Aramon and Cimarrona grape with *Botrytis cinerea* “noble rot”). The three tropical fruit wines (TF) were TF1 (frutilla), TF2 (papaya fruit and wine aging with eucalyptus chips), TF3 (mixture of raisined fruit: carambola, papaya, elder, cherries, Aramon grape, and Cimarrona grape). Finally, the two rice wines (R) were R1 (rice and fermentation with treatment of chaptalization) and R2 (roasting rice using *Aspergillus oryzae* during fermentation). Samples were stored at 6°C until analysis.

### 2.2 Physicochemical properties of fruit wines

The pH, total acidity, volatile acidity, ethanol content, and absorbances at 420 and 520 nm were determined by OIV methods (2020).

### 2.3 Determination of amino acids

Amino acids were quantified according to Gómez‐Alonso et al., (2007) and by Garde‐Cerdán et al., (2014) with slight modifications. In a 10 ml screw‐cap tube was introduced 1.75 ml of borate buﬀer 1 M (pH 9), 0.75 ml of methanol, 1 ml of sample (wine, previously centrifuged), 20 µl of internal standard (L‐2‐aminoadipic acid at 1 g L^−1^), and 30 µl of diethyl ethoxymethylenemalonate (DEEMM) and subsequently were mixed. Tubes were incubated in an ultrasound bath over 30 min and heated at 70°C in a constant temperature heater over 2 hr. The samples were cooled at room temperature for 15 min, ﬁltered using 0.45 µm PVDF syringe ﬁlters and introduced into 2 ml screw amber vials. The injection was made immediately after the ﬁltration of the samples.

A Shimadzu prominence High‐Performance Liquid Chromatograph (HPLC) (Shimadzu, Kyoto, Japan) equipped with an Automatic Liquid Sampler (ALS) and a Diode Array Detector (DAD) was used. Chromatographic separation was performed in an ZORBAX‐Eclipse plus C18 column (5 C18‐HL) (Agilent, Santa Clara, California, United States), particle size 5 µm (250 mm × 4.6 mm) thermostated at 20°C.

Two eluents, ﬁltered through a 0.45 mm Durapore^®^ membrane pore ﬁlter (Merck, Dublin, Ireland), were used as mobile phases: Eluent A: 25 mM acetate buﬀer (pH 5.8) with 0.4 g L^−1^ of sodium azide. Eluent B: 80:20 (% v/v) of acetonitrile and methanol, respectively (Sigma‐Aldrich, Madrid, Spain). Elution conditions were as follows: 0.9 ml min^−1^ ﬂow rate, 10% B during 20 min, then elution with linear gradients from 10% to 17% B in 30.5 min, maintained during 3 min, from 17% to 40% B in 31.5 min, from 40% to 72% B in 8 min, from 72% to 82% B in 5 min, from 82% to 100% B in 4 min, maintained during 3 min, from 100% to 10% B in 5.89 min, maintained during 3 min.

The injected volume of derivatized samples was 50 µl for wines. DAD was used for the detection of amino acids using the absorbance at 280 nm. In these conditions, aspartic acid (Asp), glutamic acid (Glu), asparagine (Asn), serine (Ser), histidine (His), glycine (Gly), threonine (Thr), arginine (Arg), α‐alanine (α‐Ala), γ‐aminobutyric acid (GABA), proline (Pro), tyrosine (Tyr), valine (Val), methionine (Met), cysteine (Cys), isoleucine (Ile), tryptophan (Trp), leucine (Leu), phenylalanine (Phe), ornithine (Orn), and lysine (Lys) were determined. These compounds were identiﬁed according to the retention times and UV‐Vis spectral characteristics of the derivatives of the corresponding standards. Quantiﬁcation was done using the calibration graphs of the respective standards with the same process of derivatization as the samples. The analysis was carried out in triplicate.

### 2.4 Determination of mineral contents

The mineral elements were determined using an atomic absorption spectrophotometer Shimadzu AA‐6800, (Shimadzu corporation, Japan) according to international methods of wine and must analysis (OIV, 2020). The minerals were quantified by use of standard curves of Ca (λ = 422.7 nm), Mg (λ = 285 nm), Fe (λ = 248.3 nm), Zn (λ = 213.9 nm), Pb (λ = 283.3 nm), Cu (λ = 324.8 nm), and Cd (λ = 228.8 nm).

### 2.5 Determination of total phenolic content (TPC)

Total phenolic content was performed according to the adapted Folin‐Ciocalteu method by Waterhouse (2001) with slight modifications. 10 µl of wine sample were mixed with 50 µl of Folin‐Ciocalteu reagent and 200 µl of sodium carbonate solution. The mixture was stirred for 1 min and incubated at room temperature for 30 min in the dark. The absorbance of the samples was measured at 750 nm using Perkin‐Elmer UV‐visible spectrophotometer (Lambda 25 Perkin‐Elmer Instruments, Hartford, Connecticut, EE.UU.). The TPC was expressed as milligrams of gallic acid equivalent per liter of wine sample (mg GAE·L^−1^).

### 2.6 Determination of antioxidant capacity (AC) by ABTS assay

This was performed according to the method of Re et al., (1999). 5 ml of 7 mM solution of ABTS (2, 2′‐azino‐bis‐3‐ethylbenzothiazoline‐6‐sulphonic acid) (Sigma‐Aldrich) was mixed with 88 μl of 140 mM potassium persulfate solution as stock solution and allowed to stand in the dark for 16 hr at 29°C to generate the ABTS radicals. The working solution was prepared by adding to ABTS stock solution the phosphate buffer (75 mM, pH 7.4), to an absorbance of 0.7 ± 0.03 units at 734 nm using a UV‐visible spectrophotometer (Micronal, Model B582, São Paulo, Brazil). 190 µl of the resulting blue–green ABTS radical solution was added to 10 µl of wine sample. After 10 min of incubation in dark conditions, the absorbance was read at 734 nm. Trolox (6‐hydroxy‐2,5,7,8‐tetratmethylchroman‐2‐carboxylic acid) was used as an antioxidant standard to generate the standard curve. The experiment was performed in triplicate. The results were expressed as Trolox equivalent antioxidant capacity (mg TE·L^−1^).

### 2.7 Determination of antioxidant capacity (AC) by DPPH assay

This was performed according to the method of Katalinic et al., (2004) with some modifications. A 45 mg·L^−1^ solution of DPPH (2, 2‐diphenyl‐1‐picrylhydrazyl) in methanol was prepared daily and stored in the dark. The analytical procedure was as follows: a 200 μl aliquot of extract filtered through 0.45 µm was placed in a cell and 3 ml of a 45 mg L^−1^ solution of DPPH in methanol was then added. A control sample (200 μl of water +3 ml of DPPH solution) was also prepared in parallel. Following vigorous stirring, the absorbances at 517 nm of the control sample were measured in a Beckman DU 640 spectrophotometer. The wine sample was measured under identical conditions after 30 min of incubation at room temperature. All the assays were performed in triplicate. A Trolox calibration curve was performed using concentrations between 10 and 200 mg L^−1^ Trolox. Antioxidant capacity was expressed as milligrams of Trolox equivalents per liter (mg TE L^−1^) using to calculate the inhibition percentage.

### 2.8 Statistical analysis

Multiple comparisons between means were performed by one‐way analysis of variance test (ANOVA). Homogeneous groups were calculated in order to establish significant differences between means at *p* < .05. Simple linear correlation was applied to check the relationship between ABTS and DPPH values. TPC and TAAC. In addition, principal component analysis (PCA) was used to identify the specific parameters most accurately reflecting the differences between wines. The software used was the Statgraphics Centurion (v.XVI StatPoint Technologies, Inc).

## 3 RESULTS AND DISCUSSION

### 3.1 Physicochemical properties

Table 1 showed the physicochemical properties of Cuban wines elaborated from different raw materials. The pH values of the wines ranged from 3.10 (G5) to 3.86 (TF3), optimal values to maintain wine stability and preventing them from taking place oxidative and browning reactions. Moreover, in rice wines, the pH values have a great influence on the growth and propagation of microzymes, the activity of enzyme, and the decomposition of some nutrients in fermented mash or the decomposition of yeast mesostate (Wei, 2005). In the current study, the pH values for rice wines were lower those reported in Chinese rice wines by Liu et al., (2007) and Chen and Xu (2012), ranging from 4.0 to 4.5. The total acidity varied among different wines from 9.45 (TF3) to 20.0 meq L^−1^ (G4) and the volatile acidity oscillate from 0.77 (G3) to 1.63 meq L^−1^ (TF3). The ethanol content ranged between 4.00 (G1) and 6.50% v/v (G3), excepted G5 wine which displayed the highest value 13.6% v/v. This large variation in the ethanol content in the present study is in close agreement with those obtained by Chakraborty et al., (2014) in different tropical fruit wines. However, Rupasinghe and Clegg (2007) and Chen and Xu (2012) reported higher values for fruit and rice wines (9%–14.5% v/v and 17.6%–19.4% v/v, respectively).

**TABLE 1:** Physicochemical properties of Cuban wines

Wines	Oenological parameters					
	pH	Total acidity (meq L^−1^)	Volatile acidity (meq L^−1^)	Ethanol (% v/v)	Absorbance 420 nm	Absorbance 520 nm
G1	3.49 ± 0.01^c^	14.0 ± 0.0^c^	0.96 ± 0.0^b^	4.00 ± 0.15ª	1.14 ± 0.04^b^	0.574 ± 0.03^c^
G2	3.30 ± 0.03^b^	17.0 ± 0.01^d^	1.06 ± 0.0b^c^	5.40 ± 0.21^b^	1.04 ± 0.04^b^	0.793 ± 0.02^d^
G3	3.61 ± 0.02^d^	11.0 ± 0.0^ab^	0.77 ± 0.0^a^	6.50 ± 0.35^bc^	1.34 ± 0.0^bc^	0.688 ± 0.02^cd^
G4	3.49 ± 0.03^c^	20.0 ± 0.01^e^	1.06 ± 0.0^c^	4.50 ± 0.07^a^	1.66 ± 0.02^c^	1.08 ± 0.01^e^
G5	3.10 ± 0.03ª	14.0 ± 0.01^c^	0.96 ± 0.0^b^	13.6 ± 0.0^e^	0.461 ± 0.02ª	0.204 ± 0.02^b^
TF1	3.35 ± 0.06^b^	14.3 ± 0.01^c^	1.07 ± 0.0^c^	5.70 ± 0.0^b^	0.305 ± 0.0^a^	0.085 ± 0.0^a^
TF2	3.73 ± 0.04^e^	10.5 ± 0.01ª	0.96 ± 0.0^b^	5.90 ± 0.07^b^	0.394 ± 0.04ª	0.151 ± 0.0^b^
TF3	3.86 ± 0.04^e^	9.45 ± 0.01ª	1.63 ± 0.0^e^	9.80 ± 0.0^d^	1.75 ± 0.02^c^	0.673 ± 0.02^cd^
R1	3.30 ± 0.01^b^	13.5 ± 0.01^c^	1.44 ± 0.0^d^	6.20 ± 0.07^bc^	0.365 ± 0.02ª	0.079 ± 0.01^a^
R2	3.70 ± 0.01^e^	16.4 ± 0.0^d^	0.96 ± 0.0^b^	4.70 ± 0.06ª	2.55 ± 0.04^d^	1.66 ± 0.0^e^

Note

Data are mean ± *SD* of triplicate determinations; Different superscript letters in the same column significantly different (*p *< .05).

Color is one of the main parameters of the quality of wines and has an important influence on the overall acceptability by consumers. Traditionally, the color of wines is evaluated by measuring wine absorbance at 420 nm (yellow) and 520 nm (red). The absorbance at 420 nm varied among different wines from values close to 0.305 (TF1) to 2.55 a.u. (R2). Considering this range of values, the wines were divided into two color groups namely light (<0.365 u.a.) and intense (>1.0 u.a.). The absorbance at 520 nm ranged to 0.079 (R1) to 1.66 a.u. (R2). As can be observed, R2 recorded the highest absorbance values due to rice roasted process carried out before fermentation.

### 3.2 Amino acid composition

As shown in Table 2, twenty‐one amino acids were identified and quantified in the studied Cuban wines; however, not all the amino acids are found in all wines. In this sense, aspartic acid (Asp) and glutamic acid (Glu) were detected in all wines, followed by asparagine (Asn), serine (Ser), arginine (Arg), alanine (Ala), and aminobutyric acid (GABA) detected in eight of the ten wines studied. The total amino acid content (TAAC) of grape wines ranged from 287 mg L^−1^ (G5) to 6,233 mg L^−1^ (G2) and showed significant differences at *p *< .05. The low value in G5 wine may be caused by the amino acid degradation during the process of grape dehydration by sun exposure; similar results were reported by Pereira et al., (2008) for Madeira fortified wines. In the remaining grape wines, the TAAC was higher than some reported values which ranged between 350 and 1,000 mg L^−1^ (Gutierrez‐Gamboa et al., 2020; Hernandez‐Orte et al., 2006). In general, the most abundant amino acids found in the current study for grape wines were GABA, Pro, and Tyr, accounting for 72.9% (G1), 94.3% (G2), 58% (G3), and 57% (G4) of total amino acids. Contrary, for G5 wine these amino acids were not detected. TAAC of the tropical fruit wines varied from 381 mg L^−1^ (TF2) to 1795 mg·L^−1^ (TF1) and showed significant differences at *p <* .05. The lowest TAAC was shown in Papaya wine (TF2) while “frutilla” wine had the highest content. Lee et al., (2011) reported that fresh papayas have relatively low amino acid content as compared to grape and other tropical fruits. Moreover, the amino acid profile varies significantly across the different fruits and may be influenced by a large variety of factors including variety, edaphoclimatic conditions, fertilization, management, and fermentation conditions (Clark et al., 1992; Gutierrez‐Gamboa et al., 2020). Except for papaya wine, Pro and Tyr were the most abundant amino acids in fruits wines, representing between 80.0% and 85% of total amino acid content. Regarding rice wines, it could be found that TAAC in R1 wine (620 mg L^−1^) was significantly (*p* < .05) higher than obtained for R2 wine (418 mg L^−1^). The decrease might be due to deamination and decarboxylation reactions during the roasting process (Feuillat & Charpentier, 1982). Besides, these contents were lower than 1.0 to 5.0 g L^−1^, reported in studies about Chinese rice wines (Cao et al., 2010; He et al., 2013; Shen et al., 2011). GABA and Tyr were found to be the most abundant amino acids in R1 wine, accounting 42.7% of the TAAC. In contrast, Asp and Glu were the major amino acids in R2 wine accounted for 73.4% of the TAAC.

**TABLE 2:** Amino acids contents (mg L^−1^) in Cuban wines

Amino acids	Wines									
	G1	G2	G3	G4	G5	TF1	TF2	TF3	R1	R2
Asp	20.9 ± 0.19^a^	33.0 ± 0.65^c^	93.4 ± 1.64^g^	42.3 ± 0.54^d^	193 ± 3.65^h^	28.2 ± 2.12^b^	19.8 ± 0.53^a^	60.2 ± 1.89^e^	72.4 ± 0.48^f^	201 ± 2.52^i^
Glu	32.7 ± 0.13^b^	82.9 ± 2.73^e^	81.7 ± 1.28^e^	85.5 ± 0.66^e^	15.4 ± 0.29^a^	55.3 ± 3.18^d^	57.5 ± 3.07^d^	46.8 ± 0.15^c^	31.5 ± 1.17^b^	106 ± 9.97^f^
Asn	119 ± 5.18^e^	nd	23.5 ± 1.10^bc^	32.6 ± 1.57^c^	19.9 ± 1.78^b^	27.6 ± 0.31^bc^	106 ± 28.7^d^	3.06 ± 0.01^a^	nd	20.7 ± 2.71^b^
Ser	8.74 ± 0.14^cd^	9.89 ± 3.28^d^	9.22 ± 1.04^cd^	14.7 ± 2.24^e^	nd	5.32 ± 0.23^b^	nd	7.86 ± 1.76^c^	5.25 ± 1.03^b^	3.31 ± 0.32^a^
His	nd	2.63 ± 0.62^a^	24.6 ± 0.23^d^	19.1 ± 0.42^b^	nd	nd	nd	19.8 ± 0.22^c^	nd	nd
Gly	nd	31.6 ± 3.59^c^	127 ± 2.15^e^	131 ± 3.41^f^	nd	14.1 ± 0.21^b^	7.39 ± 0.54^a^	56.6 ± 0.30^d^	31.7 ± 0.02^c^	nd
Thr	nd	11.7 ± 2.73^a^	78.1 ± 4.19^d^	56.4 ± 3.98^c^	nd	nd	nd	38.5 ± 2.20^b^	14.0 ± 1.83^a^	nd
Arg	nd	3.78 ± 0.23^a^	65.2 ± 1.16^e^	93.1 ± 3.87^f^	12.1 ± 0.03^c^	11.7 ± 1.06^c^	9.89 ± 0.02^b^	35.3 ± 0.50^d^	5.34 ± 0.01^a^	nd
Ala	nd	15.3 ± 0.05^b^	28.7 ± 0.38^e^	35.8 ± 0.93^f^	nd	39.7 ± 0.78^g^	45.8 ± 0.83^h^	19.9 ± 0.06^c^	24.1 ± 0.65^d^	6.30 ± 0.17^a^
GABA	764 ± 14.4^h^	12.6 ± 1.31^a^	101 ± 1.39^e^	198 ± 6.13^g^	nd	27.0 ± 3.14^b^	nd	48.7 ± 2.19^d^	149 ± 5.09^f^	35.6 ± 1.22^c^
Pro	nd	nd	730 ± 88.4^b^	1,490 ± 135^c^	nd	124 ± 1.98^a^	nd	128 ± 16.1^a^	nd	nd
Tyr	97.9 ± 4.85^ab^	5,864 ± 269^e^	431 ± 7.73^c^	187 ± 6.82^b^	nd	1,406 ± 41.1^d^	nd	177 ± 9.06^b^	116 ± 4.46^b^	nd
Val	11.4 ± 0.73^c^	4.60 ± 0.88^b^	4.70 ± 0.52^b^	24.3 ± 1.76^d^	nd	11.9 ± 0.00^c^	nd	3.33 ± 0.22^a^	4.85 ± 0.18^b^	nd
Met	nd	60.5 ± 7.99^d^	109 ± 1.86^e^	195 ± 5.45^f^	nd	nd	17.0 ± 0.95^a^	55.9 ± 0.70^c^	20.5 ± 1.76^a^	25.0 ± 0.84^b^
Cys	37.8 ± 0.25^e^	14.1 ± 0.52^b^	21.1 ± 0.14^c^	31.8 ± 0.06^d^	nd	nd	nd	11.5 ± 0.44^a^	nd	nd
Ile	nd	nd	2.0 ± 0.52^a^	nd	nd	nd	36.0 ± 4.84^c^	18.3 ± 1.33^b^	36.0 ± 0.11^c^	nd
Trp	65.6 ± 9.30^d^	18.9 ± 4.86^a^	nd	72.7 ± 7.10^e^	34.8 ± 0.26^b^	43.4 ± 0.77^c^	nd	nd	nd	nd
Leu	10.7 ± 0.44^a^	nd	nd	38.5 ± 3.46^b^	11.8 ± 0.55^a^	nd	nd	nd	nd	nd
Phe	nd	nd	28.2 ± 3.16^a^	129 ± 7.28^d^	nd	nd	31.8 ± 3.93^a^	36.3 ± 3.32^b^	67.8 ± 1.67^c^	nd
Orn	nd	32.0 ± 2.69^b^	113 ± 1.90^d^	235 ± 12.2^e^	nd	nd	29.0 ± 1.03^b^	47.5 ± 6.20^c^	20.6 ± 0.13^a^	nd
Lys	nd	36.9 ± 2.44^b^	103 ± 6.14^d^	153 ± 11.0^e^	nd	nd	21.0 ± 0.91^a^	56.7 ± 1.02^c^	21.1 ± 0.13^a^	20.0 ± 0.73^a^
TAAC	1,169 ± 12.6^e^	6,233 ± 242^i^	2,174 ± 122^g^	3,265 ± 96.2^h^	287 ± 1.30^a^	1795 ± 50.3^f^	381 ± 36.1^ab^	872 ± 34.4^d^	620 ± 8.64^c^	418 ± 13.7^b^

Note

Data are mean ± *SD* of triplicate determinations; Different superscript letters in the same line significantly different (*p *< .05).

Overall, Tyr was found to be the highest in G2, TF1, and TF3 wines; this amino acid has been known to play a role in the wine taste (acerbity) if it presents at high levels (Cao et al., 2010). Besides, Pro was the most abundant amino acid found in G3 and G4, consistent with the findings of Gutierrez‐Gamboa et al., (2020) and Wang et al., (2014) who reported this as major amino acid in Tempranillo and Cabernet Sauvignon grape wines. Moreover, Asp was the major amino acid in G5 and R2 wines. Cao et al., (2010) also reported of high levels of Asp in Chinese rice wines, which contributes with umami flavor to these wines. Finally, GABA was the predominant amino acid in G1 and R1 wines and Asn in TF2 wine. Recent studies have also shown that there is a large amount of GABA in Chinese rice wine. This amino acid, catalyzed from glutamic acid by glutamic acid decarboxylase, is the most important inhibiting neurotransmitter in the brain with numerous health effects (Joye et al., 2011; Wu, Xu, Long, Wang, et al., 2015). Likewise, Giovanni et al., (2015) reported high levels of Asn and GABA amino acids in Italian wines.

### 3.3 Mineral composition

Table 3 showed the mineral composition of the wines studied. A total of seven mineral elements (Ca, Mg, Fe, Zn, Pb, Cu, and Cd) were determined and the total mineral contents (TMC) were calculated. As can be observed, the TMC ranged from 162 mg L^−1^ in TF3 wine (raisined fruits) to 424 mg L^−1^ in G5 wine (botrytised and raisined grapes) and exhibited significant differences (*p* < .05) for most of the Cuban wines them. Ca and Mg are defined as macroelements and in the current study were found to be the most abundant minerals, representing more than 95% of the TMC, in all wines (Paneque et al., 2010; Shen et al., 2013). Fe and Zn were among the minor mineral present, while Pb, Cu, and Cd were below the detection limits in all wine types and, therefore, below the maximum acceptable limits for these elements reported by OIV (OIV, 2020). These results in the current study are important because these last‐mentioned metals can become highly toxic if they accumulate in biological system (Rupasinghe & Clegg, 2007). The TMC of different grape wines varied from 170 mg L^−1^ (G1) to 424 mg L^−1^ (G5), and there were no significant differences between G1 and G4 wines. The TMC ranged to 162 mg L^−1^ (TF3) to 308 mg L^−1^ (TF2) in tropical fruit wines showed significant differences among all wines (*p* < .05). For rice wines, TMC was significant different varied from 176 mg L^−1^ (R1) to 246 mg L^−1^ (R2), lower than that reported by Qian et al., (2019) in Chinese rice wines.

**TABLE 3:** Minerals contents (mg L^−1^) in Cuban wines

Wines	Mineral elements							
	Ca	Mg	Fe	Zn	Pb	Cu	Cd	TMC
G1	86.6 ± 8.57^ab^	78.4 ± 12.8^bc^	3.84 ± 1.55^d^	0.58 ± 0.18^d^	˂0.15	˂0.05	˂0.01	170 ± 10.6^ab^
G2	116 ± 6.39^c^	94.1 ± 4.22^e^	5.35 ± 0.15^f^	1.21 ± 0.08^g^	˂0.15	˂0.05	˂0.01	218 ± 10.8^c^
G3	196 ± 6.89^e^	83.6 ± 5.34^cd^	10.2 ± 0.52^g^	1.01 ± 0.12^f^	˂0.15	˂0.05	˂0.01	291 ± 0.90^e^
G4	94.8 ± 3.04^ab^	80.3 ± 6.20^bcd^	4.81 ± 0.12^ef^	0.83 ± 0.06^e^	˂0.15	˂0.05	˂0.01	181 ± 3.23^b^
G5	369 ± 43.0^g^	53.0 ± 16.2^a^	1.58 ± 0.89^c^	0.13 ± 0.11^a^	˂0.15	˂0.05	˂0.01	424 ± 25.7^g^
TF1	158 ± 0.04^d^	88.7 ± 0.08^de^	4.33 ± 0.01^de^	0.79 ± 0.01^e^	˂0.15	˂0.05	˂0.01	252 ± 0.03^d^
TF2	223 ± 18.9^f^	79.9 ± 1.00^bcd^	4.09 ± 0.88^de^	0.39 ± 0.05^bc^	˂0.15	˂0.05	˂0.01	308 ± 20.9^f^
TF3	83.0 ± 5.29^a^	76.7 ± 5.20^bc^	1.35 ± 0.08^bc^	0.31 ± 0.01^b^	˂0.15	˂0.05	˂0.01	162 ± 2.4^a^
R1	104 ± 1.57^bc^	71.2 ± 7.75^b^	0.55 ± 0.02^a^	0.45 ± 0.02^c^	˂0.15	˂0.05	˂0.01	176 ± 6.14^b^
R2	94.9 ± 3.12^ab^	150 ± 5.66^f^	0.63 ± 0.01^ab^	0.49 ± 0.0^cd^	˂0.15	˂0.05	˂0.01	246 ± 2.53^d^

Note

Data are mean ± *SD* of triplicate determinations; Different superscript letters in the same column significantly different (*p* < .05).

Regarding the macroelements minerals individually, Ca levels (369 mg L^−1^) were the highest in G5 wine (botrytised and raisined Aramon and Cimarrona grapes) and were significantly higher (*p* < .05) than in all other studied wines. Contrary, Ca content was the lower in G1, G4, TF3, and R2 wines without significant differences among them. In addition, Mg contents were the highest in the previous wine mentioned R2 (raw rice and roasted rice, 150 mg L^−1^), exhibiting significant differences with the wines in the current study (*p* < .05). Moreover, this rice wine was the only one that showed a higher content of Mg than Ca. This is in accordance with the results pointed by Shen et al., (2013), for Chinese rice wines. On the contrary, this mineral presented the lowest content (53.0 mg L^−1^) in G5 wine. In the current study, the concentration of microelements Fe and Zn ranged between 0.55 (R1)‐10.2 mg L^−1^ (G3) and 0.13 (G5)‐1.21 mg L^−1^ (G2), respectively. It is known that Fe, Zn, and Cu are closely related to the winemaking process (Rupasinghe & Clegg, 2007; Shen et al., 2013), and this fact in addition with the different raw material used in the elaboration of Cuban wines, could explain the variability of the concentrations of these elements.

### 3.4 Total phenolics content and antioxidant capacity

The TPC of different Cuban wines was between 200 mg GAE L^−1^ in TF1, TF2 and R1 (*p* < .05) to 2,250 mg GAE L^−1^ in R2 (Table 4). This wide range is due to concentration and composition of the phenolic compounds present in wines depends largely on the source of raw material and the winemaking processes. Based on the TPC and according to Rupasinghe and Clegg (2007), studied wines can be categorized into three major groups: high TPC (R2 wine, 2,250 mg GAE L^−1^); moderately high TPC (G3 and G4 wines, ranged from 817 to 904 mg GAE L^−1^, respectively); and low TPC (G1, G2, G5, TF1, TF2, TF3, and R1 wines, ranged from 195 to 480 mg GAE L^−1^).

**TABLE 4:** Total phenolic content (TPC) and antioxidant capacity (AC) in Cuban wines

Wines	TPC (mg GAE L^−1^)	AC	
		ABTS assay (mg TE L^−1^)	DPPH assay (mg TE L^−1^)
G1	480 ± 9.08^e^	1602 ± 12.5^d^	298 ± 1.44^e^
G2	377 ± 7.26^c^	1,208 ± 100^c^	200 ± 1.48^d^
G3	817 ± 9.98^f^	2,176 ± 28.4^e^	294 ± 0.67^e^
G4	904 ± 31.8^g^	2,519 ± 28.4^f^	294 ± 1.39^e^
G5	287 ± 8.17^b^	537 ± 24.1^b^	170 ± 7.03^c^
TF1	195 ± 9.08^a^	417 ± 29.2^ab^	103 ± 7.01^c^
TF2	197 ± 0.91^a^	326 ± 5.43^a^	66.0 ± 1.84^b^
TF3	426 ± 96.2^d^	1,277 ± 59.1^c^	259 ± 5.37^e^
R1	214 ± 2.72^a^	377 ± 27.2^a^	14.7 ± 2.16^a^
R2	2,250 ± 1.82^h^	2,526 ± 15.8^f^	292 ± 1.13^e^

Note

Data are mean ± *SD* of triplicate determinations; Different superscript letters in the same column significantly different (*p* < .05).

It can be seen that the TPC of the different types of rice wine is significantly different (*p* < .05) from each other, and it indicated that these contents in these wine types are mainly derived from the treatment given to the raw material. Resulting in drastically different wine quality parameters under the same fermentation conditions which agrees with the results reported by Cai et al., (2019) and Chay et al., (2020) in Chinese and Cambodia rice wines, respectively.

The results indicated that Cuban tropical fruit wines contain lower levels of phenolic compounds as compared to elderberry, cherry, blueberry, and black currant wines which contain high phenolic content averaging between 1509 and 2005 mg GAE L^−1^ (Pantelic et al., 2014; Rupasinghe & Clegg, 2007). However, they were close to the values reported by Rupasinghe and Clegg (2007) for pear, peach, and apple wines, which were between 310 and 451 mg GAE L^−1^. The TPC of TF3 sample was significantly higher (*p *< .05) than the other tropical fruit wines due to the enrichment with grape must during its elaboration.

The highest values of TPC were found in wines R2, G4, and G3 (2,250, 904, and 817 mg GAE·L^−1^, respectively), which were elaborated using fungus during their winemaking. Some authors observed a decrease in total phenolic compounds of botrytized grapes compared with healthy grapes; however, they reported an increase in flavan‐3‐ol family (Carbajal‐Ida et al., 2016). The studied wines were elaborated from different raw materials and different winemaking procedures, for example, the use of chips, so the differences in the values of total phenolic compounds could be due to these facts.

The antioxidant capacity of food is determined by the presence of different antioxidants, with different mechanisms of action; therefore, the antioxidant capacity of food products should be evaluated with a variety of methods which occur with different mechanisms (Moo‐Huchin et al., 2014). The highest concentration values by ABTS assays were obtained in G4, G3, and R2 wines, reached values higher than 2,000 mg TE·L^−1^. The two first were red wines elaborated added *Botrytis fungus*, and the third was from roasting rice with *Aspergillus oryzae*. Meanwhile, the highest values by DPPH assay (>290 mg TE·L^−1^) were found in above‐mentioned wines in addition to G1, this last wine was elaborated with skin contact which increases the phenolic compounds concentration. Contrary, R1 and TF2 presented the lowest values of antioxidant capacity measured by both assays.

To establish a correlation between antioxidant capacity and the bioactive composition, the correlation coefficients were determined (Table 5). The antioxidant capacity measured by DPPH assay expresses a low correlation with TPC (*R* = 0.3473) and a moderate positive correlation was found for the values obtained by ABTS assay (*R* = 0.6200) correlated with phenolic compounds. In this sense, some authors found a high correlation between the phenolic content and the antioxidant capacity (Kuskoski et al., 2005; Reddy et al., 2010), while others found no relationship (Imeh & Khokhar, 2002; Thaipong et al., 2006). The results suggest that the phenolic compounds may be not the unique contributors to the antioxidant capacity in the Cuban wine studied in this research. In the other hand, the correlation of total amino acid content with the antioxidant capacity values showed a low correlation. In all case analyzed, the correlation was not significant at *p* < .05 level. Therefore, the antioxidant capacity in Cuban wines can be attributed to the actions of different antioxidant compounds, such as phenolic compounds, amino acids, or bioactive peptides among other substances such as isothiocyanates, vitamins, and flavonoids. whose effect can be antagonistic or synergistic.

**TABLE 5:** Correlation coefficients for total phenolic compounds (TPC) and total amino acid content (TAAC) with the antioxidant capacity

	Correlation coefficients	
	DPPH assay	ABTS assay
TPC	0.3473*	0.6200*
TAAC	0.0470*	0.0561*

* Not significant at *p* < .05 level.

**FIGURE 1:**
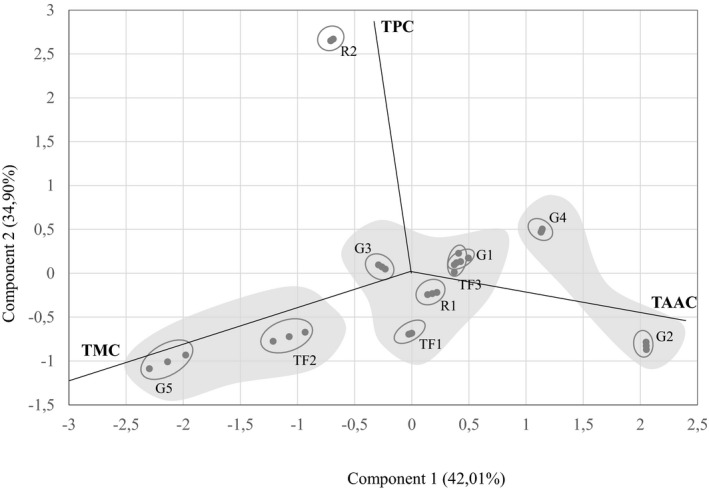
Principal component analysis: biplot representation of wines and statistical variables. G1 = botrytised Aramond grape/Cimarrona grape; G2 = Cimarrona grape; G3 = botrytised Aramond grape; G4 = botrytised and concentrated Aramond grape/Cimarrona grape; G5 = Aramond grape; TF1 = frutilla; TF2 = papaya; TF3 = Aramond grape/Cimarrona grape/mixture of fruits (frutilla, papaya and carambola); R1 = rice; R2 = roasting rice/Aspergillus oryzae. TAAC, total amino acids content; TMC, total mineral contents; TPC, total phenolic compound

To identify the specific parameters most accurately reflecting the differences between wines, the TPC (total phenolic content), TAAC (total amino acid contents), and TMC (total mineral contents) data for the different Cuban wines were subjected to multivariate principal component analysis. Figure 1 shows the scores of each sample on the plane defined by the first two principal components (PC) (eigenvalue >1), which accounted for 76.91% of the total variance and allowed the different wines elaborated from several raw material to be discriminated. Based on the results, the PC1 accounted for 42.01% of the total variance and correlated positively with total amino acid contents and negatively with total mineral contents and being the variables with the highest statistical weights on this component. The variable total phenolic compounds were that exerting more influence on the PC2, which accounted for 34.90% of the total variance, showing a positive correlation with this component. Except for samples corresponding to R2, no statistical separation could be clearly observed for the other wines, mainly because of the high dispersion in the data. However, taking to account scores on the PC1, 3 wine groups could be defined. The first, for the wines G5 and TF2, the variable with the highest statistical weight on the PC1 was total mineral content (TMC), these wines showed the highest values in this parameter. Other groups include the wines G4 and G2, and in this case, the variable with the highest statistical weight was the total amino acid content (TAAC). Finally, the third group includes the rest of the wines, and they did not show a clear separation and obtained low scores with respect to all the variables.

## 4 CONCLUSION

In summary, Cuban wines elaborated from different raw materials as follows: grapes, tropical fruits, and rice and through different winemaking processes were found to be a source of essential amino acids, minerals, and phenolic compounds. The significantly highest values of total amino acid content and total mineral content were displayed in G2 wine and G5 wine, respectively. Ca and Mg were the principal mineral elements quantified in all wines. Excepted R2 wine, Ca presented higher levels than Mg. Regarding the total phenolic content, rice wines were significantly highest. Among all wines, grape wines (G1, G3, and G4) and R2 wine showed the highest value of antioxidant capacity. The multivariate principal component analysis carried out allowed define three wine groups influenced by PC1, in this variable total mineral content and total amino acid content had the highest statistical weight. Therefore, the results of the studied wines must be taken into consideration to improve the winemaking processes and nutritional quality of the Cuban wines.

## CONFLICT OF INTEREST

The author declares that there is no conflict of interest that could be perceived as prejudicing the impartiality of the research reported.

## AUTHOR CONTRIBUTION

**Lazaro Nuñez:** Investigation (equal); Writing‐original draft (equal). **María P. Serratosa:** Supervision (equal); Writing‐original draft (equal); Writing‐review & editing (equal). **Ana Godoy:** Formal analysis (equal); Investigation (equal). **Laura Fariña:** Formal analysis (equal); Investigation (equal). **Eduardo Dellacassa:** Investigation (equal); Supervision (equal). **Lourdes Moyano:** Methodology (equal); Supervision (equal); Writing‐original draft (equal); Writing‐review & editing (equal).
